# Violent delinquency in a Brazilian birth cohort: the roles of breast feeding, early poverty and demographic factors

**DOI:** 10.1111/j.1365-3016.2009.01091.x

**Published:** 2010-01

**Authors:** Beatriz Caicedo, Helen Gonçalves, David A González, Cesar G Victora

**Affiliations:** a Postgraduate Program in Epidemiology, Federal University of PelotasPelotas, Brazil; b University of Antioquia, National School of Public HealthMedellin, Colombia

**Keywords:** breast feeding, juvenile delinquency, violence, poverty, race, Pelotas cohort

## Abstract

Caicedo B, Gonçalves H, González DA, Victora CG. Violent delinquency in a Brazilian birth cohort: the roles of breast feeding, early poverty and demographic factors. *Paediatric and Perinatal Epidemiology* 2010; **24:** 12–23.

We investigated the association between breast feeding, economic factors and conviction for violent delinquency by age 25 years among subjects of the 1982 Birth Cohort from Pelotas, Southern Brazil. Information on breast-feeding pattern and duration was collected in childhood, during the 1983, 1984 and 1986 follow-ups. Information on socio-economic and family characteristics was also obtained between 1982 and 1996. Of the 5914 livebirths enrolled in the cohort, 5228 had obtained an identification document within the state of Rio Grande do Sul, and could thus be identified in judiciary databases. The outcome studied was conviction due to a violent act between ages 12 and 25 years. A total of 106 young people had been convicted at least once (3.0% of men and 1.0% of women). Subjects born to black or mixed mothers and coming from low-income families were at higher risk of having been convicted. Neither crude nor adjusted analyses showed any association between breast feeding and conviction for violent delinquency. Violent delinquency apparently depends more on social factors than on individual factors such as breast feeding.

## Introduction

Urban violence has increased markedly in Brazil in the last two decades, with a growing participation of young people as perpetrators of violent delinquency – i.e. acts offensive to the judicial order in force and subject to punishment by legal authorities.^1^,^2^ According to violence surveys, acts of delinquency are being committed at increasingly younger ages, with perpetrators returning to crime later in life in an increasingly violent manner.^3^ Cohort studies from different sociocultural settings indicate that the early onset of violence and delinquency is associated with more severe and chronic violence in adulthood.^4^–^6^ According to these studies, approximately 35% of children with a history of antisocial behaviour become adult delinquents, and about 5% of these are responsible for approximately 50% of crimes considered as the most severe by society, including robbery, armed assault and rape.^6^ Even though these individuals represent only a small fraction of the population, the consequences of their acts are not small, as attested by the physical and emotional sequelae commonly found among their victims.^7^,^8^

Violent behaviour is the cumulative result of the interaction of many factors – individual, family, community and societal.^4^,^9^ For instance, factors such as pregnancy complications, maternal depression, child rejection, parental criminality and delinquency, parental conflict, severe and inconsistent disciplinary practices, and lack of parental supervision have been found to increase the risk of committing a violent offence.^10^–^13^

Social inequality and inequity also play an important role in the aetiology of violent delinquency.^14^,^15^ Concentration of wealth, unemployment and uncertainty with respect to the future can also increase the risk.^15^,^16^ Most studies investigating the role of socio-economic conditions show that individuals engaging in violent delinquency come from low-income families, but that other social and environmental factors such as poor academic performance, higher population density, migration or parental absence can also be predictive of delinquency.^16^–^18^

Skin colour has been identified as an important predictor of both violent behaviour and arrest or prosecution.^19^ Prior studies have shown that young Blacks are more frequently arrested and convicted because of violent behaviour than young Whites by the Brazilian justice system.^20^ Whether these differences are due to socio-economic, cultural or other factors is not clear and this needs to be investigated.

In addition to these factors, certain authors suggest that breast feeding may be protective against the development of violent behaviour. The mechanisms for this association would include biological factors, such as the presence in breast milk of substances that contribute to neuronal development, or behavioural and emotional factors – connected to the act of breast feeding – that could inhibit the later emergence of delinquent or antisocial behaviour.^21^,^22^ There is abundant evidence for the important role played by breast feeding in child development, especially with regards to its short- and long-term effects in the prevention of infectious diseases.^23^,^24^ However, very few studies have investigated the protective effect of breast feeding on the aetiology of delinquent behaviour. A search of the literature returned only two studies on this subject, both resulting from the analyses of a birth cohort study begun in 1977 in New Zealand. The first publication evaluated the relationship between breast feeding and conduct disorders (such as shyness, hyperactivity, and social isolation) among children between 6 and 8 years of age, detecting no statistically significant associations.^25^ The second study analysed the relationship between duration of breast feeding and antisocial and/or delinquent behaviour at age 18 years, also failing to detect any significant associations.^26^

Even though evidence regarding these relationships is still insufficient and widely questioned,^22^ findings pertaining to long-term effects are highly sensitive to the effects of other associated variables, especially those of a socio-economic nature, which can distort the magnitude of such associations.^23^ Mothers who breast feed are known to display distinct socio-economic and behavioural characteristics from those who do not. These characteristics may influence an individual's behaviour at different times during life, interfering with his or her probability of engaging in violent delinquency.^23^,^27^ From this perspective, longitudinal studies provide an important contribution to the understanding of different factors that influence violent delinquency. In addition to allowing for the topic to be addressed in regard to current social and family settings, such studies are also capable of investigating current behaviour as a result of past events. There are no published studies addressing this subject in Brazil, nor are there investigations of other potential early determinants of delinquency, especially delinquency involving violence and subject to judicial punishment. Surveys on this subject may aid the formulation of public policies and programmes aimed at violence prevention.

The goal of the present study was to evaluate the effect of breast feeding, early poverty and demographic factors on violent delinquency resulting in judicial conviction among young people participating in a birth cohort. To achieve this objective we tested the following main hypothesis: that breast feeding protects against violent behaviour and that childhood poverty increases the risk of such behaviour. As secondary hypotheses, we postulated that the following factors would increase the risk of conviction due to violence: male sex, low birthweight, significant neonatal morbidity, being born to a teenage or single mother, being exposed to smoking during pregnancy, high birth order and separation of the parents in the first 3 years of life.

## Methods

The city of Pelotas is located in the state of Rio Grande do Sul, in the extreme south of Brazil, and has an estimated population of 364 thousand.^28^ In January 1982 a birth cohort was launched by enrolling all 5914 hospital deliveries taking place in the urban area of Pelotas.^29^ By 2006, nine follow-up visits had been carried out, which have allowed us to investigate the influence of perinatal, demographic, environmental, nutritional and care-related variables on the health of participating subjects.^30^ A detailed account of the methods of this cohort study is provided elsewhere.^29^,^30^ For the present study, the information on all cohort members who were issued a mandatory identity card in the state of Rio Grande do Sul was obtained from the files maintained by the Department of Public Security and Justice. The state identification number allows the investigation of all crimes committed by cohort members in several municipal and state data sources. The search was carried out between July 2007 and March 2008.

**Figure 1:**
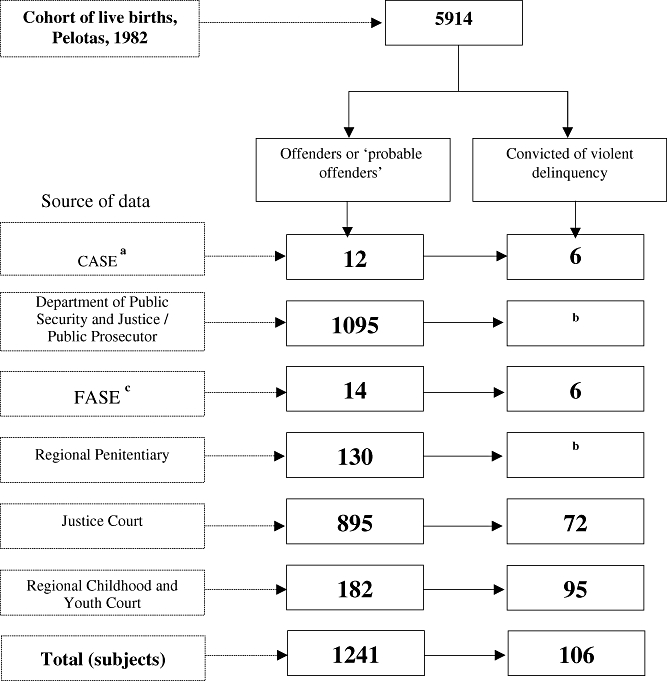
Number of subjects of the 1982 cohort with criminal records and convicted of violent delinquency up to age 25 years (2008). ^a^Pelotas Juvenile Detention Centre; ^b^This source does not provide information on conviction; ^c^State Socio-Educational Care Foundation. Note: The Rio Grande do Sul State Department of Public Security and Justice and the Pelotas Public Prosecutor provide criminal records [*Boletim de Ocorrência Criminal*]. The Regional Penitentiary provides data on individuals caught in the act by the Civil Police and on those under preventive detention until a judicial sentence is awarded.

Seven data sources were accessed (Fig. 1): (1) the Pelotas Juvenile Detention Centre (CASE), (2) the Department of Public Security and Justice of the state of Rio Grande do Sul, (3) the Pelotas Public Prosecutor's Office, (4) the state Socio-Educational Care Foundation (FASE) for adolescents, (5) the Pelotas Regional Penitentiary, (6) the state Court of Justice, and (7) the Pelotas Regional Childhood and Youth Court. Violent crimes resulting in conviction were the outcome for the analyses, given the severe legal nature of such acts. Judicial conviction is characterised by the attribution of legal responsibility for a delinquent act to an individual. Data on all convictions were collected from the Pelotas Regional Childhood and Youth Court, which maintains records of convictions for young people aged 12–21 years, and from the State Court of Justice for conviction of subjects at ages 18 years and above. Data from the state Department of Public Security and Justice and from the Pelotas Regional Penitentiary provided information on ‘probable offenders’ of crimes among cohort members aged 12–25 years committed across the entire state. The ‘probable offender’ category included young people considered by the judicial system as indicted, accused, perpetrators, suspects or teenage offenders.

An agreement made with these seven institutions ensured the confidentiality of subject identification and the type of use of judicial data in analyses and publications. The research project was approved by the Research Ethics Committee of the Federal University of Pelotas School of Medicine.

All crimes retrieved from cohort members' criminal records were classified as *violent* or *non-violent*. Intentional physical injury, robbery, intentional homicide, attempted homicide, rape, robbery followed by homicide, or kidnapping for ransom were considered as violent acts. All cohort members carrying judicial convictions for any of the forms of violence listed above were subsequently classified as convicted for violent delinquency. Remaining subjects – including those convicted for other criminal activities, those not yet convicted at the end of the search period, and/or those lacking criminal records – were classified as not having been convicted for violent delinquency.

Data on duration of breast feeding were reported by mothers in 1983, 1984 and 1986, when mothers were interviewed regarding the child's dietary pattern and duration of breast feeding. In order to avoid recall bias with respect to time and duration of breast feeding, we used the response provided in the follow-up closest to the date of weaning. The dietary pattern of children during the first year of life was classified as *predominant* for children breast fed with or without supplementation with other liquids (tea or water), but who did not receive solid or semi-solid foods, and *partial* for children who received breast milk complemented by other milks (cow's milk or formula) or other dietary complements.^31^ Age at weaning was defined as age of total interruption of breast feeding (predominant or partial), categorised in six groups according to time in months (<1; 1–2.9; 3–5.9; 6–8.9; 9–11.9; ≥12).

Individual and familial risk factors in the first years of life were included in the analysis as potential confounders. At the time of birth, maternal skin colour was self-referred by the mother and grouped into white and black/mixed. Skin colour of cohort members is not available for the entire sample, and therefore maternal skin colour was used. However, there is a strong correlation between maternal skin colour reported by the mother at time of delivery (in 1982) and that self-reported by the subject at 23 years of age (in 2005). We observed that 89.7% of cohort members born to white mothers considered themselves white, while 90.3% of those born to black or mixed mothers considered themselves as black or mixed.

In 1982, we also collected the following maternal characteristics: age (<20 or 20+ years), marital status (with or without partner), schooling (0–4; 5–8; ≥9 years at school), family income in number earning minimum wages (<1; 1.1–3; 3.1–6; >6), smoking during pregnancy (yes/no), obstetric complications (yes/no) and low birthweight (yes/no). Toxaemia, diabetes, pre-eclampsia, eclampsia, hypertension and maternal haemorrhage were considered as obstetric complications. During the 1986 follow-up, we collected another two variables that were used in the present analysis, the number of older and younger siblings (0, 1, ≥2).

stata 9.0 software (Stata Corp., College Station, USA) was used for data analysis. For univariable analysis, we sought associations using the chi-squared test, adopting a significance level of 20% (*P* < 0.20) to identify potential confounders. Multivariable analyses were carried out using Poisson regression based on a conceptual model taking into account a proposed hierarchy of causal relationships. The most distal determinant was maternal skin colour, which may influence all other variables in the model, but is not influenced by them. The second level included the effects of maternal socio-economic, demographic and behavioural variables (age, marital status, family income, maternal schooling and smoking during pregnancy). The third level included the effects of low birthweight, obstetric complications, and number of younger and older siblings. Finally, in the last level, we examined the effect of duration of breast feeding. For the analysis of nominal variables we used the chi-squared test for heterogeneity. For ordinal variables, such as duration of breast feeding – for which there was a hypothesis of dose–response effect, we used the linear trend test. Because women were markedly less likely than men to present violent behaviour,^18^ all analyses were stratified by sex.

## Results

Of the 5914 livebirths in the urban area of Pelotas in 1982, 5228 cohort members were identified who had a civil record (identification data, with or without criminal records) in the database of the Department of Public Security and Justice. Of these, 2873 had at least one criminal record. The most frequent reasons for inclusion in the registry were being a victim (39.1%), being a ‘probable offender’ (23.7%) or being a witness of a crime (8.8%).

In total, 277 males and 55 females belonging to the cohort were convicted of a crime by 25 years of age. Of these, 106 (81 males and 25 females) were convicted of at least one violent act: seven of homicide, 66 of physical injury, 37 of robbery, kidnapping for ransom, or robbery followed by homicide, and six of rape. The largest proportion had their first conviction between 17 and 21 years of age (42.5%); one-quarter were convicted at age 21 or older. Figure 1 summarises findings from each source. It should be noted that the records provided by the state Department of Public Security and Justice and by the Regional Penitentiary did not allow us to estimate the proportion of cohort members convicted of violent delinquency, given that some individuals in the records were registered as ‘probable offenders’. The total number of records from the various sources exceeds the total number of subjects with one or more record, given the duplication that exists between sources.

**Figure 2:**
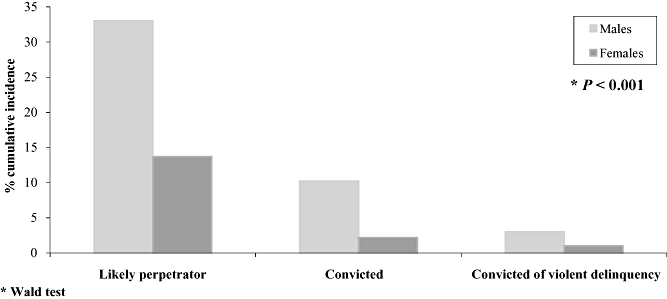
Cumulative incidence of different types of criminal record based on judicial sources according to sex. Pelotas cohort, Southern Brazil, 1982–2008.

Figure 2 shows that, for all cases of probable offence or conviction, cumulative incidence up to age 25 is higher among males than among females. Cumulative incidences of probable offence and conviction were 33.0% [95% confidence interval [CI] 31.3, 34.8] and 10.2% [95% CI 9.0, 11.3], respectively. Among females, these were 13.7% [95% CI 12.3, 15.0] and 2.2% [95% CI 1.6, 2.8], respectively. Cumulative incidence of violent delinquency was 3.0% [95% CI 2.3, 3.6] among males and 1.0% [95% CI 0.6, 1.4] among females.

Unadjusted analysis of male subjects (Table 1) showed that cumulative incidence of conviction for violent delinquency decreased significantly as family income increased. Subjects born to women of black or mixed skin colour were twice as likely to have been convicted of violent delinquency as those born to white women (*P* = 0.01). Subjects with two younger siblings by the age of 4 years showed a significant increase in convictions (*P* = 0.005). There was no linear trend for conviction with respect to duration of breast feeding, either predominant or partial. Mother's age and marital status, smoking during pregnancy, low birthweight and number of older siblings were not associated with the outcome. However, maternal schooling was inversely associated with the outcome. Conviction was present for 3.9% of males born to women with 0–4 years of education, compared with only 1.1% of males whose mothers had 9 years or more of school (*P* = 0.001).

**Table 1:** Unadjusted and adjusted analysis of the association between individual, maternal, and pregnancy-related variables and conviction for violent delinquency up to age 25 years among males from the 1982 Pelotas Birth Cohort

		Lifetime conviction for violent delinquency					
		Unadjusted			Adjusted^a^		
Level	Independent variables	*n*	CI%	CIR [95% CI]	*P*^b^	CIR [95% CI]	*P*^b^
1	Mother's skin colour				0.01		0.01
	White	2228	2.6%	1.0 Reference		1.0 Reference	
	Black or mixed	489	4.7%	1.8 [1.1, 2.9]		1.8 [1.1, 2.9]	
2	Mother's age (years)				0.2		0.5
	<20	399	4.0%	1.4 [0.8, 2.5]		1.2 [0.7, 2.1]	
	≥20	2320	2.8%	1.0 Reference		1.0 Reference	
	Mother's marital status				0.7		0.9
	With husband or partner	2516	2.9%	1.0 Reference		1.0 Reference	
	Without husband or partner	201	3.5%	1.2 [0.6, 2.5]		0.9 [0.4, 2.1]	
	Family income (no. of minimum wages)				*P* < 0.001^c^		*P* < 0.001^c^
	≤1	542	3.9%	12.2 [1.6, 90.0]		10.6 [1.4, 78.8]	
	1.1–3	1337	3.7%	11.5 [1.6, 83.1]		10.7 [1.5, 77.2]	
	3.1–6	515	1.9%	6.1 [0.8, 47.4]		5.9 [0.8, 46.1]	
	>6	314	0.3%	1.0 Reference		1.0 Reference	
	Smoking during pregnancy				0.3		0.6
	No	1761	2.7%	1.0 Reference		1.0 Reference	
	Yes	958	3.4%	1.3 [0.8, 2.0]		1.1 [0.7, 1.8]	
3	Obstetric complications				0.8		0.9^d^
	No	2506	3.0%	1.0 Reference		1.0 Reference	
	Yes	213	3.3%	1.1 [0.5, 2.4]		1.1 [0.5, 2.4]	
	Low birthweight				0.7		0.5^d^
	No	2546	3.0%	1.0 Reference		1.0 Reference	
	Yes	172	3.5%	1.2 [0.5, 2.7]		1.3 [0.6, 3.1]	
	Number of younger siblings				0.005^c^		0.01^c^,^d^
	0	1649	2.3%	1.0 Reference		1.0 Reference	
	1	627	4.3%	1.9 [1.2, 3.0]		1.9 [1.2, 3.0]	
	≥2	79	5.1%	2.2 [0.80, 6.0]		1.9 [0.7, 5.4]	
	Number of older siblings				0.6^c^		0.8^c^,^d^
	0	1044	2.8%	1.0 Reference		1.0 Reference	
	1	653	2.9%	1.1 [0.6, 1.9]		1.1 [0.6, 2.0]	
	≥2	658	3.2%	1.2 [0.7, 2.0]		1.1 [0.6, 1.9]	
4A	Duration of partial breast feeding (months)				0.2^c^		0.1^c^,^d^,^e^
	<1	585	1.5%	1.0 Reference		1.0 Reference	
	1–2.9	670	4.2%	2.7 [1.3, 5.7]		2.6 [1.2, 5.7]	
	3–5.9	580	2.9%	1.9 [0.9, 4.2]		2.0 [0.9, 4.8]	
	6–8.9	249	2.0%	1.3 [0.4, 3.9]		1.3 [0.4, 4.2]	
	9–11.9	108	0.9%	0.6 [0.1, 4.7]		0.9 [0.1, 7.4]	
	≥12	382	4.4%	2.9 [1.3, 6.4]		2.9 [1.3, 6.6]	
4B	Duration of predominant breast feeding (months)				0.1^c^		0.06^c^,^d^,^f^
	<1	656	1.8%	1.0 Reference		1.0 Reference	
	1–1.9	383	4.2%	2.3 [1.1, 4.8]		1.6 [0.7, 3.5]	
	2–2.9	448	2.0%	1.1 [0.5, 2.6]		1.1 [0.5, 2.6]	
	3–3.9	658	3.3%	1.8 [0.9, 3.7]		1.7 [0.9, 3.5]	
	≥4	294	4.1%	2.2 [1.0, 4.9]		2.2 [1.0, 4.6]	
	Total	2719	3.0%				

a Adjusted for all variables in the same level or in higher levels with *P* < 0.2.

b Wald test for heterogeneity.

c Wald test for linear trend.

d Also adjusted for maternal schooling in 1982.

e Model fitted with partial breast feeding as dependent variable.

f Model fitted with predominant breast feeding as dependent variable.

CI, confidence interval; CI%, cumulative incidence; CIR, cumulative incidence ratio.

Adjusted analyses (Table 1) were carried out according to the hierarchical levels of causality. Among males, the effects of maternal schooling and family income showed significance levels ranging from 7% to 10% in adjusted analysis, compared with their highly significant effects in the unadjusted analyses. This resulted from collinearity between these two variables, which are intimately associated. In such cases, it is appropriate to present only one of these variables in the adjusted model, in this case family income which, in unadjusted analysis, was more strongly associated with the outcome. With the exclusion of maternal schooling, the *P* value for family income in the adjusted model was 0.001. Data for maternal education are not presented in the tables but due to the possibility of residual confounding (the *P* level for schooling was 0.1) we opted to adjust the analyses of variables in levels 3–4 for education as well as income.

Cumulative incidence ratios (CIRs) for children of poor families remained high when compared with the reference category. The effect of the number of younger siblings also remained significant. The unadjusted effect of predominant or partial breast feeding was virtually unaltered by adjustment. There was no dose–response relationship between duration of breast feeding and risk of conviction for violent delinquency.

In the analysis restricted to females (Table 2), low birthweight was not included in the model, since none of low birthweight had been convicted. Unadjusted analyses showed that subjects born to adolescents or to mothers of black or mixed skin colour had a higher risk of conviction for violent delinquency than those born to adult (*P* = 0.001) or white (*P* = 0.05) mothers. Convictions for violent delinquency decreased significantly as family income and mother's schooling increased. For maternal schooling the risk of conviction was 0.3% in the group with nine or more years, compared with 1.6% in the group with 0–4 years (data not shown in the table). Mother's marital status, smoking during pregnancy, obstetric complications, number of older and younger siblings, and breast feeding were not significantly associated with the outcome.

**Table 2:** Unadjusted and adjusted analysis of the association between individual, maternal, and pregnancy-related variables and conviction for violent delinquency up to age 25 years among females from the 1982 Pelotas Birth Cohort

		Lifetime conviction of violent delinquency					
		Unadjusted			Adjusted^a^		
Level	Independent variables	*n*	CI%	CIR [95% CI]	*P*^b^	CIR [95% CI]	*P*^b^
1	Mother's skin colour				0.05		0.05
	White	2077	0.8%	1.0 Reference		1.0 Reference	
	Black or mixed	431	1.9%	2.3 [1.0, 5.2]		2.3 [1.08, 5.2]	
2	Mother's age (years)				0.001		0.01
	<20	374	2.7%	3.8 [1.7, 8.4]		2.9 [1.3, 6.4]	
	≥20	2134	0.7%	1.0 Reference		1.0 Reference	
	Mother's marital status				0.4		0.9
	With husband or partner	2323	0.9%	1.0 Reference		1.0 Reference	
	Without husband or partner	184	1.6%	1.7 [0.5, 5.7]		1.1 [0.3, 3.4]	
	Family income (no. of minimum wages)				<0.001^c^		0.01^c^
	≤1	503	1.8%	14.4 [1.8, 113.5]		9.2 [1.1, 74.4]	
	1.1–3	1186	1.3%	10.2 [1.4, 77.1]		7.9 [1.1, 59.6]	
	3.1–6	806	0.1%	1.0 Reference		1.0 Reference	
	Smoking during pregnancy				0.9		0.6
	No	1631	1.0%	1.0 Reference		1.0 Reference	
	Yes	878	1.0%	1.0 [0.5, 2.4]		0.8 [0.4, 1.8]	
3	Obstetric complications				0.9		0.8
	No	2304	1.0%	1.0 Reference		1.0 Reference	
	Yes	205	1.0%	1.0 [0.2, 4.1]		1.2 [0.3, 5.1]	
	Number of younger siblings				1.0^c^		0.6^c^
	0	1554	1.0%	1.0 Reference		1.0 Reference	
	1	564	0.9%	0.9 [0.3, 2.3]		0.7 [0.3, 1.8]	
	≥2	60	1.7%	1.6 [0.2, 12.0]		1.1 [0.1, 8.5]	
	Number of older siblings				1.0^c^		0.2^c^
	0	912	1.0%	1.0 Reference		1.0 Reference	
	1	658	1.1%	1.1 [0.4, 2.9]		1.7 [0.6, 4.6]	
	≥2	608	1.0%	1.0 [0.4, 2.8]		1.9 [0.6, 5.7]	
4A	Duration of partial breast feeding (months)				0.6^c^		0.5^c^,^d^
	<1	479	0.8%	1.0 Reference		1.0 Reference	
	1–2.9	640	1.2%	1.5 [0.5, 4.9]		1.4 [0.4, 4.6]	
	3–5.9	562	0.5%	0.6 [0.1, 2.8]		0.7 [0.2, 3.0]	
	6–8.9	216	0.9%	1.1 [0.2, 6.0]		1.3 [0.3, 6.8]	
	9–11.9	90	2.2%	2.7 [0.5, 14.3]		3.0 [0.6, 15.6]	
	≥12	401	1.2%	1.5 [0.4, 5.5]		1.4 [0.4, 5.2]	
4B	Duration of total breast feeding (months)				0.5^c^		0.7^c^,^e^
	<1	532	1.1%	1.0 Reference		1.0 Reference	
	1–1.9	304	0.3%	0.3 [0.0, 2.4]		0.3 [0.0, 2.4]	
	2–2.9	483	1.9%	1.7 [0.6, 4.6]		1.8 [0.6, 5.0]	
	3–3.9	646	0.6%	0.6 [0.2, 1.9]		0.6 [0.2, 2.2]	
	≥4	301	0.7%	0.6 [0.1, 2.9]		0.6 [0.1, 3.0]	
	Total	2509	1.0%				

a Adjusted for all variables in the same level or in higher levels with *P*< 0.2.

b Wald test for heterogeneity.

c Wald test for linear trend.

d Model fitted with partial breast feeding as dependent variable.

e Model fitted with predominant breast feeding as dependent variable.

CI, confidence interval; CI%, cumulative incidence; CIR, cumulative incidence ratio.

The effect of mother's age on conviction among females remained significant in adjusted analysis (Table 2). Daughters of adolescent mothers were three times more likely to have been convicted of violent delinquency when compared with daughters of adult mothers. Family income remained as a major risk factor. There was no association between duration of breast feeding, either predominant or partial, and conviction for violent delinquency. As for males, the unadjusted effect of mother's schooling disappeared after adjustment (*P* = 0.4).

Additional analyses were carried out to explore possible pathways between skin colour and conviction. After adjustment for family income, the effect of skin colour decreased markedly, being no longer statistically significant (CIR for black males fell from 1.8 in unadjusted analysis to 1.5 after adjustment for family income; for females, this reduction was from 2.3 to 1.6).

All the analyses for both males and females were repeated, treating breast feeding as dichotomous variables considering different cut-off points (ever vs. never breast fed; ≤1 vs. >1 month; ≤6 vs. >6 months; below vs. above the median), but the lack of association with convictions remained.

Finally, analyses of breast feeding and convictions were stratified by skin colour (white vs. black/mixed) and family income (<3 vs. 3 or more minimum wages) but the lack of association remained within all subgroups (data not shown). We tested interactions between breast-feeding duration (<3 or 3+ months) and family income, skin colour, mother's age and number of siblings. None of these interactions had a *P* value below 0.2.

## Discussion

This study was aimed at investigating the possible impact of breast feeding and early sociodemographic conditions on conviction for violent crime in young persons. Contrary to our hypothesis, we found that total or predominant duration of breast feeding was not associated with conviction.

Subjects born to poor families and those born to black or mixed mothers were more likely to have been convicted. These results agree with the existing literature.^19^,^20^ Because previous analyses of this cohort showed that black or mixed mothers are also more likely to breast feed for 9 months or longer,^32^ the possibility of residual confounding was explored. Subgroup analyses for white and for black/mixed mothers confirmed the lack of a protective effect for breast feeding in both groups. Thus, our results do not support our initial hypothesis – that breast feeding may protect against violent behaviour problems throughout life.

Comparison of our results with those in the literature is problematic, due to lack of consistency in outcome definitions. Only the cohort study carried out in New Zealand evaluated conviction for violent delinquency using a similar definition. Convictions were investigated up to age 18 years, and were self-reported. This study did not find significant differences in terms of incidence of conviction between breast fed and non-breast fed subjects.^26^

Some authors who analysed the relationship between delinquency and breast feeding from the biological standpoint argue that the high concentrations of long-chain polyunsaturated fatty acids present in breast milk would play a fundamental role in the neurological development of the infant,^32^ helping to mould its response to situations of emotional stress which require impulse control.^33^,^34^ There is evidence in the literature that breast feeding may have an effect on the quality of bonding between mother and infant, possibly offsetting adverse environmental influences leading to delinquent behaviour.^34^ However, both mechanisms have been disputed by other authors, who consider them as insufficient to establish a causal relationship.^35^,^36^ In any case, it is difficult to separate, on the basis of epidemiological data, the biological or psychosocial effects of breast feeding. A recent randomised clinical trial in Belarus contributes to this debate.^37^ When evaluating the long-term effects of breast feeding on behavioural problems among children at 6.5 years of age (emotional problems, hyperactivity and prosocial behaviour), that study found no evidence of child relationships.^37^ Our results are compatible with these observations. The negative findings reported here should be weighed against the well-known benefits of breast feeding in both the short and long term. For example, the long-term effects of breast feeding on cognitive outcomes and school performance have been widely documented, both in this cohort as well as elsewhere.^38^–^40^

A recurring issue with observational studies of the long-term consequences of breast feeding is confounding by socio-economic status. In high-income countries, better-off women are more likely to breast feed than the poor.^41^ There is a weak association between breast feeding and socio-economic status in our sample. Rich mothers are more likely to start breast feeding and to breast feed until 6–9 months. Thereafter, poor mothers are more likely to continue breast feeding for 1 year and beyond.^32^ The lack of a clear pattern of association between breast feeding and socio-economic status makes confounding unlikely. This argument is supported by the fact that there was little difference between the crude and adjusted coefficients for breast feeding, and by the finding that stratified analyses (by income and skin colour) failed to demonstrate a significant effect of breast feeding in any subgroup.

Our findings confirm the well-known difference in convictions for violent delinquency between men and women. Most studies of criminality exclude females from their analyses due to low statistical power.^11^,^42^ For these authors, female misconduct is manifested through apparently non-violent behaviours, such as alcohol and drug abuse, diet-related conditions, depression and early pregnancy.^43^ However, as in males, our results suggest that skin colour and family income in females are related to conviction for violent delinquency.

Family income was the socio-economic characteristic most strongly associated with violent delinquency in both sexes. This effect remained after adjustment for mother's skin colour. In summary, subjects who were poor as young children showed a high risk of being convicted of violent delinquency, a result which is compatible with previous literature.^44^,^45^ When young the poor may seek to ascend socially or to overcome specific economic difficulties by practicing activities considered as illegal.

Being born to a black or mixed mother doubled the risk of conviction for violent delinquency. Skin colour of cohort members was collected only after age 18, and therefore, was not available for all subjects included in the present analyses. Other authors have reported that blacks face great difficulty in social ascension. However, inequity and ethnic/racial discrimination may also play a relevant role in the Brazilian judiciary system.^20^,^46^ Generally speaking, black or mixed individuals may be perceived as potential delinquents, with consequent increase in probability of conviction.^20^ Given that the effect of skin colour declined markedly and became non-significant after adjustment for mediating factors such as income, we can infer that high incidence among black subjects is due largely to poverty, highlighting the difficulties encountered by the poor when dealing with the legal system.^47^

Having two or more younger siblings at age 4 years was a significant risk factor among males. It is possible that parental care and supervision of older children are compromised in these families. It is also a possibility that older siblings, when they grow up, are placed in a position of being partly or entirely responsible for family maintenance, and consequently suffer from deprivations or frustrations that may have affected their social behaviour.

Family and environmental influences are apparently related to the development of criminal behaviour in the young. One study in Arizona showed that mothers with many children were less able to supervise their children.^48^ Families with larger numbers of children tend to be poor and live in places with high levels of criminality, with easier access to weapons, drugs and alcohol. All these factors increase the risk of criminal behaviour.^18^ In addition, families with younger, unmarried and uneducated mothers, with three or more children, face stressful situations on a daily basis. This would reduce their ability to educate and provide proper discipline for their children and to solve family conflicts appropriately. This last condition has been highlighted as an important risk factor for violent criminal behaviour.^18^ Therefore, there is a range of situations related to family structure, maternal characteristics and parent–child relationships that could contribute to delinquency in young people.^17^,^18^

We also found greater risk of delinquency among daughters of adolescent mothers. This association has been previously reported.^44^,^49^ According to the literature, this may not be a causal effect, that is, having an adolescent mother may be a marker for other predictive factors for conviction for violent delinquency. Poor young women are known to be more likely to become mothers than better-off adolescents, which in turn may affect their chances of economic ascension and increase the frequency of behavioural problems among their children.^50^ On the other hand, it is possible that the observed effect is direct: for example, an undesired pregnancy during adolescence may lead mothers to be less tolerant with their children, resorting to physical punishment as an educational practice, which may, in its turn, influence the child's behaviour later in life.^49^,^50^

The fact that some associations were significant only among males (number of younger siblings) or only among females (mother's age) cannot be explained based solely on our data. There is a need for replication in other settings as well as for qualitative studies on possible reasons for these differences.

Several studies have indicated that certain characteristics – such as absence of a partner at the time of delivery,^50^ smoking during pregnancy,^11^ obstetric complications^10^,^51^ and low birthweight,^52^,^53^ increase the risk of violent delinquency. The lack of an association in the 1982 cohort may be related to the low number of cases exposed to such characteristics (Tables 1,2). However, the small magnitude of the measure of effect (CIR) suggests that these variables do not have an important role in our population, regardless of statistical power.

It is important to consider the limitations of the present study. The databases accessed contained discrepancies regarding the spelling of the names of some of the subjects, which may have prevented their identification, thereby reducing the final number who were identified – nevertheless 88.4% of all cohort members were located. Second, any violent delinquency committed outside Rio Grande do Sul State was not included in the analyses. In the 2005 follow-up visit to the cohort, about 75% of all subjects were still living in Pelotas, and most of the remainder lived in other large cities in the state, so that the proportion committing crimes elsewhere is likely to be small. It is also known that only a small fraction of crimes are proven and punished.^54^ Therefore, our analyses may have been affected by classification errors, and the true incidence of delinquency is underestimated. A further limitation concerns the relatively young age of cohort members. It is likely that a number of people in the cohort may yet undertake acts of violent delinquency leading to conviction in the future. Finally, the variables studied were restricted to those obtained at an early age. Other important risk factors for violence – especially those pertaining to family relationships – are not available for analysis. In spite of these drawbacks, however, this is the first study to assess the effects of breast feeding on violent behaviour in a middle-income country. The use of a longitudinal, prospective design reduced the possibility of recall bias with regards to duration of breast feeding as well as to confounding factors.

The present study highlighted factors that may be measured at an individual level, stressing the importance of socio-economic conditions as determinants of behavioural problems among young people. Addressing these factors and taking steps to change them, including measures to close the gap between the rich and poor and to ensure equitable access to goods, services and opportunities may be key points of intervention for dealing with violent behaviour. In addition, early detection of risk factors for violent delinquency may ensure that such young people have a greater chance of social mobility, and thus represent one of the building blocks of a comprehensive strategy against the growing epidemic of violence in our society.

Our findings suggest that violent delinquency, both sporadic and persistent, may be related to a range of factors which include individual, biological, family-related and social features. Violent delinquency seems to depend more on social factors than on individual factors such as breast feeding.
